# A survey for the readiness of Greek midwives for the adoption of evidence-based practice (EBP)

**DOI:** 10.18332/ejm/128270

**Published:** 2020-11-20

**Authors:** Angeliki Ladopoulou, Dimitrios Charos, Elissavet Maniatelli, Irene Plassara, Paraskevi Giaxi, Victoria G. Vivilaki

**Affiliations:** 1 Department of Midwifery, University of West Attica, Athens, Greece; 2 Second Department of Obstetrics and Gynecology, Aretaieion Hospital, National and Kapodistrian University of Athens, Athens, Greece

**Keywords:** evidence-based practice (EBP), evidence-based practice readiness survey (EBPRS), midwifery

## Abstract

**INTRODUCTION:**

The holistic approach of healthcare practice in midwifery demands the use of evidence-based practice (EBP) in all aspects of clinical care. Applying EBP in every day healthcare practice by midwives offers various significant benefits. The aim of the present study was to investigate and assess the knowledge and awareness of midwives in Greece with regard to EBP.

**METHODS:**

Data collection took place from October 2012 to January 2013 among midwifery staff within two national ‘urban’ healthcare hospitals of Athens and the department of midwifery in the Athens Technological Institute. The sample consisted of 209 participants of which 109 were midwives and 100 student midwifes. Both were invited to complete a questionnaire specifically designed for the study.

**RESULTS:**

Only 43.5% of midwives declared awareness of the term EBP, while 36.4% had to search for general evidence about twice a month in order to support their role. The first source of information to support clinical practice was found to be ‘asking colleagues’ (52.2%) followed by ‘internet search in general’ (48.8%), but not in the EBP databases. In addition, 61.2% of respondents stated that EBP would definitely contribute to the provision of better quality midwifery care.

**CONCLUSIONS:**

For a successful implementation of EBP, it is required initially to train personnel to develop their abilities, to provide information on the way to use different data sources and encourage midwifery personnel to take initiatives and be part of the decision-making process.

## INTRODUCTION

There is a continuous struggle nowadays by healthcare organizations, governments and academics to improve the standards of the healthcare services provided. Consequently, more and more healthcare professionals turn towards providing the newest and best possible care practices. Promoting the use of EBP is definitely a turn in that direction^1-4^.

Evidence based practice (EBP) includes: use of the best available research documents from medical care professionals in clinical practice; the values of the patient^5,6^; clinical expertise^5^; the preferences of the patient^5-6^; and pathophysiological knowledge^6^.

The use of EBP is widely considered a paradigm shift, and imposes a strong change in the way midwives perform their everyday tasks^5,12,13^. It is actually a shift from opinion-based clinical decision-making to evidence-based decision-making^3,7,8^. EBP, in essence, consists of the best scientific evidence and clinical expertise combined with the skill and ability to acknowledge and express the wishes and desires of the patients^9^. EBP as a process, when employed efficiently, does provide better care not only in the area of midwifery but also in general health practice altogether, as it uses the most recent research evidence data and applies them to clinical practice. In recent years, the importance of supporting EBP has received increased emphasis and today is considered as the model of healthcare^3,8^.

EBP aims at the best results in the care of patients as well as in more economical and effective care^10^. The term ‘evidence-based practice’ (EBP) has been incorporated into the notions of both researchers and midwives from the early 1970s till now^3,12^. According to Hunter^13^ , and Owens and Kennedy^8^, a midwife should be a ‘critical thinker’ who will use safe and effective practices for the benefit of the women and their newborns. Therefore, midwives who care the most about women and their neonates do believe nowadays that the best care is achieved only through the use of evidence in order to manage treatment-related decisions more effectively^2,4^.

In midwifery we are actually fortunate because in some areas of our practice we already have high quality evidence of interventions associated with beneficial outcomes for women and/or their infants^7^. This is a privilege that must not remain unutilized. Especially young midwifery professionals that are more accustomed to accessing EBP-related online resources must definitely take advantage of this knowledge in order to support their clinical role, strengthen their position and authority, and ultimately gain confidence to manage normal pregnancy and childbirth in partnership with women^1^. The fact that EBP has also been incorporated into higher education curricula is very important as well, it will hopefully boost the awareness on young medical professionals, and is an effective way to help students to learn to think critically^8,9,14^.

As stated above, the ability for clinical thinking is an indispensable part of EBP implementation, and its development should be an objective for the academics and the clinical teachers of midwifery^8,15^.

In 2018, The Technological Educational Institute of Athens was renamed to the University of West Attica, upgrading in this way the existing high standards of midwife education. The Midwifery students are admitted to the University through exams at national level or qualifying examinations, and the duration of studies is four years.

The new midwifery curriculum at the University of West Attica, is adapted to modern developments of Midwifery Science, with the main feature of scientifically substantiated theoretical knowledge and gradual building of the clinical application and experience, starting in the first year of study.

Accordingly, it is important that higher institutions always strive for the most effective approach to teaching students the knowledge and skills required for EBP, so that upon commencing clinical practice they can confidently incorporate research evidence into their clinical decisionmaking^16^.

The aim of the present survey was to study, derive knowledge and also ascertain the opinions of midwives about EBP in clinical practice. More specifically, the aims include the following: 1) test a Greek version of the evidence-based practice readiness survey (EBPRS) and assess its reliability and validity in measuring in EBP working environment in a sample of midwives in Athens, and 2) examine the factor structure of the Greek EBPRS.

## METHODS

### Sample and data collection

**Figure 1:**
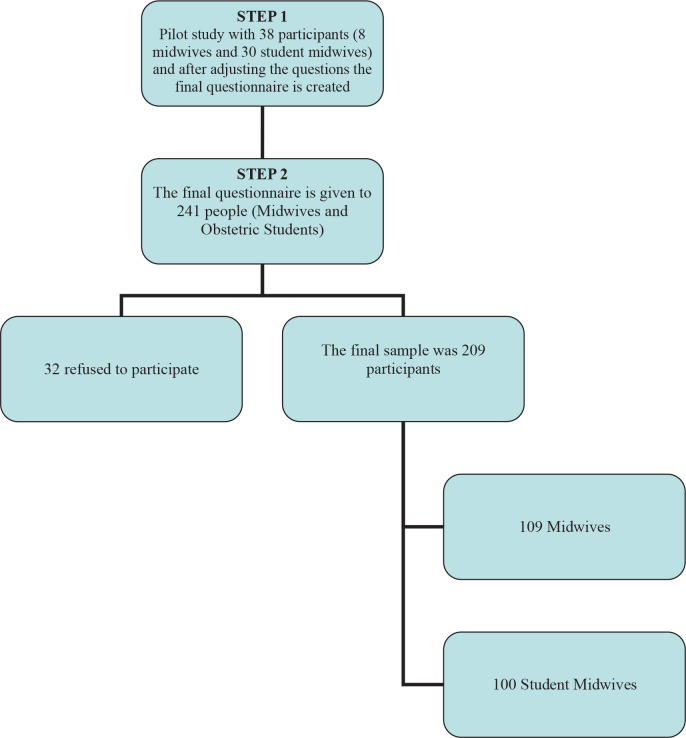
Flow chart of the research procedure for the Greek EBPRS

Data collection took place from October 2012 to January 2013 among midwifery staff within two national ‘urban’ healthcare hospitals of Athens and the department of midwifery in the Athens Technological Institute. The sample collection was based on a stratified data collection methodology. Inclusion criteria were: fluency in the spoken and written Greek language, willingness to participate, and completion of at least one year of study. In total, 241 midwives were approached and 209 agreed to participate (response rate: 87%), of which 100 were midwives and 109 student midwives (Figure 1). Midwives and student midwives were encouraged to discuss any concerns they might have and were informed that the managing midwife of the hospital would be informed of their responses to the screening. All participants were informed verbally about the results of working conditions of midwives.

### Pilot study

In a pilot study, the new Greek version of the EBPRS was tested with 38 participants, of whom 8 were graduate midwives and 30 were student midwives. As part of the cultural adaptation process, in-depth interviews were conducted to test respondents’ understanding of scale items. Participants gave their views about the clarity of each item, the relevance of the content to their situation, the comprehensiveness of the instructions, and their ability to complete the Greek version EBPRS on their own.

### Instrument

The EBPRS is an 80-item self-reported scale consisting of statements describing midwives’ EBP readiness, and is divided into five domains including: Informational needs, EBP-knowledge, EBP-attitude, Workplace culture, and EBP-assessments. The ‘Informational needs’ domain contained 35 items of the informational literacy for evidence-based midwifery practice C questionnaire with various rank-order formats^17^. Some items required respondents to answer ‘more than adequate’, ‘adequate’, ‘less than adequate’, or ‘totally inadequate’ to questions about workplace informational resources, such as online resources. The intent of these items was to identify at what frequency and in which way midwives prefer to seek information in order to support their professional role.

The ‘Workplace culture’ domain used the six items of midwifery evidence-based practice survey C, which measures the EBP-culture in a unit or organization^18^. The ‘Perceived EBP-knowledge’ domain designed by Thiel and Ghosh^19^ aimed to identify the midwives’ perception of having enough knowledge, skills and access to resources to undertake EBP. The perceived knowledge scale consisted of eight items and had a five-point Likert-type scale, measuring the level of agreement–disagreement (1=strongly disagree to 5=strongly agree) with each statement. The ‘EBPattitude’ domain is based on midwives’ attitudes toward EBP scale (MATES), which was developed to examine midwives’ attitudes and beliefs toward EBP^19^.

The 17-item MATES is based on a five-point Likert-type scale and measures the level of agreement–disagreement (1=strongly disagree to 5=strongly agree). Examples of MATES statements are the following: ‘There is no reason for me personally to adopt evidence-based practice because it is just a “fashion” that will pass with time’, ‘Evidence-based practice ignores the “holistic” aspect of midwifery’, and ‘Evidence-based practice must be implemented to achieve desired patient outcomes’.

Finally, the ‘EBP-assessments’ domain used 14 items aimed to identify the way midwives’ assess EBP, based on a five-point Likert-type scale and measured the level of agreement–disagreement (1=strongly disagree to 5=strongly agree).

### Translation and validation process

The original version of the EBPRS was translated into Greek using the back-translation strategy for cross-cultural research. Two experienced bilingual translators performed a forward translation from the original English version independently, one was an advanced practice midwife and the other a physician (Step 1).

Both forward versions were then conciliated and incorporated into the Greek version by an expert panel (Step 2) using a consensus procedure. Back translation was carried out by an English teacher who understood the Greek language but had no knowledge of the EBPRS or access to the original version in English (Step 3). The semi-final version was derived from a reconciliation of the original, back, and forward translations. As this was in agreement with the English original, the translation was considered to be correct (Step 4).

### Cultural adaptation

The translated tool was culturally adapted through a cognitive debriefing process that was used to identify any language problems and to assess the level of respondents’ understanding of the items’ content. During this stage, the reconciled Greek version of the EBPRS was pilot-tested among 8 graduate midwives and 30 student midwives, who fulfilled the following criteria: students participating should have completed at least one year of study, Greek speaking, and willing to participate. As part of the cultural adaptation process, in-depth interviews were conducted with the participants in order to reveal any misconceptions. The participants were encouraged to point out any incomprehensible parts of the questionnaire and to suggest translation alternatives. Finally, participants’ comments were included in the final Greek version of EBPRS.

The approval for translation and use of the tool was granted by the original author of the EBPRS^19^. The study protocol was approved by the research ethics boards of the five national healthcare hospitals. All participants entering the study provided written informed consent after receiving a complete description of the study and having the opportunity to ask for clarifications. Along with the questionnaires was a cover letter explaining the purpose of the study, providing the researchers’ affiliation and contact information, and clearly stating that answers would be confidential and anonymity would be guaranteed in the final data reports.

### Statistical analysis

Statistical analysis was performed using SPSS v. 20.0 for windows (IBM SPSS Statistics 20.0, Chicago, IL, USA, 2011), and STATA 12 for the confirmatory factor analysis (CFA). Descriptive characteristics (including means, standard deviations, frequencies, and percentages) and the assumptions of normality, homogeneity, and independent cases of the sample were checked. The normality assumption was checked using the skewness and kurtosis values. In particular, for skewness, absolute value should be >3 and, for kurtosis, absolute value should be >10 (Kline^20^). Statistical significance was set at p<0.05.

### Psychometric properties of the Greek EBPRS

#### Internal consistency

Internal consistency and reproducibility (test-retest reliability) were measured as part of the reliability analysis of the translated instrument. Internal consistency was determined by Cronbach’s alpha, Spearman-Brown coefficient and Guttman split-half coefficient^21^. McNemar and Cohen’s kappa were calculated for individual items^22^.

#### Factor structure

The underlying dimensions of the scale were checked with an exploratory factor analysis (EFA) using a varimax rotation and principal components method, as is the usual descriptive method for analyzing grouped data^23^. Principal component analysis with varimax rotation was conducted to determine the dimensional structure based on: 1) an eigenvalue >1; 2) variables load >0.50 on only one factor and on other factors <0.40; 3) meaningful interpretation of factor structure; and 4) an accurate scree plot^24^ with means of communalities above 0.60.

Computations were based on a covariance matrix, as all variables were receiving values from the same measurement scale^25^. During factor analysis, a Bartlett’s test of sphericity (p<0.05) and a Kaiser-Meyer-Olkin (KMO) that measured sampling adequacy of 0.699, were also implemented. A factor was considered important if its eigenvalue was >1, with factor analysis identifying 7 independent subscales. Cronbach’s alpha was carried out on each subscale, to highlight how items grouped together^26^.

## RESULTS

### Sample characteristics

Table 1 presents the demographic characteristics of the respondents (n=209). The majority of the participants were female (96.2 %, n=126) and aged <30 years (60.3 %, n=126). Nearly half of the participants (47.8%, n=100) were students of the Department of Midwifery at the Athens Technological Institute, having successfully completed the first year of study while the rest were graduates of the department (52.2%, n=109), many of whom had postgraduate diploma courses, an MSc (12.4%, n=26). The majority of the respondents had work experience of 0–5 years (61.3%, n=128). Descriptive characteristics of the Greek EBPRS are shown in Table 2. The communalities for the Greek EBPRS are presented in Table 3.

**Table 1:** Sociodemographic characteristics of the Greek midwives as well as the student midwives in the study period 2012–2013

*Characteristics*	*Midwives*	*Student midwives*	*Total*
	*n (%)*	*n (%)*	*n (%)*
**Age** (years)			
<30	26 (12.4)	100 (47.8)	126 (60.3)
30–39	39 (18.7)	-	39 (18.7)
40–49	31 (14.8)	-	31 (14.8)
50–59	13 (6.2)	-	13 (6.2)
**Gender**			
Male	7 (3.3)	1(0.5)	8 (3.8)
Female	102 (48.8)	99 (47.4)	201 (96.2)
**Postgraduate education**			
None	179 (85.6)	-	179 (85.6)
Master’s	26 (12.4)	-	26 (12.4)
PhD	1 (1.4)	-	1 (1.4)
**Years of work**			
0–5	28 (13.3)	100 (87.7)	128 (61.3)
6–10	28 (13.3)	-	28 (13.3)
11–20	27 (12.9)	-	27 (12.9)
>21	26 (12.5)	-	26 (12.5)

**Table 2:** Descriptive statistics of items of the Greek EBPRS in the study period 2012–2013 among Greek midwives and student midwives

Items*		*Mean*	*SD*	*Skewness*	*Kurtosis*
**Informational needs**					
**13**	I search the databases	2.47	1.221	0.502	-0.576
**18**	I have ability to search in CINAHL	3.84	1.302	-0.883	-0.202
**19**	I have ability in searching MEDLINE	3.39	1.434	-0.389	-1.094
**29**	I search in CINAHL	3.96	1.526	-1.123	-0.351
**30**	I use MEDLINE/PUBMED	3.53	1.641	-0.594	-1.328
**Workplace culture**					
**36**	There is a good deal of teamwork among nursing personnel	2.89	1.203	0.357	-0.319
**37**	They are satisfied with the interaction among the nursing staff	3.16	1.178	0.001	-0.122
**38**	Physicians in general cooperate with nursing staff	3.30	1.373	-0.096	-0.295
**39**	There is a lot of nurse-doctor teamwork on my unit	3.22	1.420	-0.142	-0.589
**40**	There is ample opportunity for nursing staff to participate in decision-making	3.59	1.429	-0.065	-0.785
**41**	They have all the voice they want in planning policies and procedurals for the unit	3.67	1.477	-0.093	-0.889
**EBP-attitude (MATES)**					
**42**	Personally, I have no reason to adopt EBP, as it is a fashion which will fade	3.61	1.135	-0.718	0.277
	away				
**44**	EBP ignores clinical experience	3.38	0.984	-0.433	0.254
**46**	EBP ignores the ‘art’ of midwifery	3.46	1.096	-0.648	0.659
**49**	EBP ignores the holistic approach of midwifery	3.31	1.136	-1.280	2.082
**50**	EBP must be implemented to achieve desired patient outcomes	2.18	0.884	0.658	0.461
**53**	Midwives in general shouldn’t apply EBP, given the fact that midwifery has to do with women and not with statistics	3.45	1.130	-0.682	0.240
**54**	I am confident that I can engage in EBP	2.74	0.862	-0.418	1.131
**55**	I have enough abilities as not to be obliged to deal with EBP	2.87	0.974	-0.745	1.631
**56**	EBP ignores the patients’ values	3.52	1.043	-0.790	1.106
**57**	Using EBP for nursing increases the certainty that patient outcomes will be met	2.33	0.936	0.326	0.352
**58**	It’s important that our hospital adopts practice based on indications in midwifery practice	2.62	1.112	-0.099	-0.260
**EBP-knowledge**					
**60**	I’m well aware about research in midwifery from talks with my colleagues	2.79	1.080	0.174	-0.351
**61**	I have convenient access to nursing research journals	2.76	1.079	-0.114	-0.683
**65**	Midwives such as clinical instructors act as mentors in my department	2.74	1.039	0.156	-0.179
**66**	I can read a nursing research report and make a judgment about its scientific merit	2.80	0.874	0.045	0.335
**EBP-assessments**					
**67**	EBP contributes to more economical midwifery care	2.48	0.779	-0.344	-0.414
**68**	EBP in dispensing economical resources in a more effective way	2.55	0.713	-0.282	0.294
**69**	EBP increases the efficiency of midwifes	2.34	0.812	-0.112	-0.105
**70**	EBP offers to health units scientific patentability	2.16	0.887	-0.232	0.192
**71**	The power of habit and routine in clinical practice block the application of EBP	2.37	0.958	0.052	-0.398
**72**	The lack of confidence in the ability of applying new clinical methods makes the application of DBP more difficult	2.38	0.904	0.404	-0.062
**73**	I feel sufficiently confident to apply EBP in midwifery care	2.70	0.876	-0.206	0.572
**74**	I have the knowledge to apply EBP in clinical practice	2.83	0.985	-0.806	1.323
**75**	I’m confident enough to evaluate the condition of the women I care	2.15	0.921	0.596	0.787
**76**	I’m confident in the methods I apply in medical care	2.03	0.828	0.245	0.425
**77**	I’m in a position to evaluate the validity of the data of a research	2.51	0.899	-0.302	0.710
**78**	I’m in a position to apply the new research data in clinical practice	2.63	0.822	-0.376	0.713
**79**	The evaluation of my clinical work is directly associated with EBP	2.75	0.965	-0.478	0.418

* Only loading of >0.50 are presented. Greek language version of the table is given in the Supplementary file, Table S1.

**Table 3:** Exploratory factors and explained variance after rotation for the Greek EBPRS in the study period 2012–2013 among Greek midwives and student midwives

*Factors*	*Rotation sums of squared loadings*						
	*Items*	*Rescaled loadings*	*Eigen values*	*% of variance*	*Cumulative variance*	*Cronbach’s alpha*	*Standardized alpha*
**Factor 1 (EBP-attitude)**		3.383	7.175	36.925	0.561	0.535	
	42	0.777					
	44	0.670					
	46	0.680					
	49	0.827					
	50	0.562					
	53	0.484					
	54	0.364					
	55	0.424					
	56	0.441					
	57	0.512					
	58	0.910					
**Factor 2 (Informational needs)**			7.884	16.718	16.718	0.828	0.826
	13	0.673					
	18	1.022					
	19	1.201					
	29	1.271					
	30	1.329					
**Factor 3 (Workplace culture)**			6.146	13.032	29.750	0.813	0.815
	36	1.042					
	37	0.901					
	38	1.071					
	39	0.666					
	40	1.254					
	41	1.295					
**Factor 4 (EBP-knowledge)**			2.564	5.437	42.368	0.493	0.495
	60	0.618					
	61	0.586					
	65	0.476					
	66	0.366					
**Factor 5 (EBP- assessments)**			2.021	4.286	46.648	0.767	0.773
	67	0.564					
	68	0.385					
	69	0.444					
	70	0.471					
	71	0.402					
	72	0.502					
	73	0.564					
	74	0.657					
	75	0.645					
	76	0.507					
	77	0.505					
	78	0.531					
	79	0.472					

The majority (36.4 %) of respondents indicated that they occasionally (1–2 times a month) needed information to support their professional role. They preferred to search for information by reading articles or books (62.2%) and attending congresses, seminars or training programs (55.5%). In general, most of the respondents stated that they were not familiar with the term EBP (56.5%) and even more said they had not received instructions on how to use computer databases (CINAHL, Medline) (65.6%). Also, a large number of participants (31.6%) perceived that they had enough knowledge to participate in the EBP and a further large proportion (64.4%) said that it was not certain whether or not they possessed such a knowledge list.

Also, 69.4% of respondents agreed that there was a good level of teamwork among midwives, and 44% were satisfied with the level of interaction between the midwifery staff.

### Factor structure

#### Exploratory factor analysis (EFA) and confirmatory factor analysis (CFA)

The KMO measure of sampling adequacy for the EBPRS was 0.699, while Bartlett’s test of sphericity was statistically significant (χ^2^=2920, n=156, df=741, p<0.001), indicating that the matrix was fit for performing the analysis.

The rotated loading matrix estimated with maximum likelihood indicated that the item ‘I have ability in internet search’ had a very low weight in the second factor and therefore it was eliminated. Removing the item ‘I have ability in internet search’ from the CFA significantly improved model fit. Thus, this item was excluded from scoring the Greek version of the EBPRS for the purposes of the study. Therefore, following the criteria described in Methods, only 23 items were used in the following analysis.

### Validity

#### Face and content validity

The Greek version of EBPRS was well accepted by the midwives. It was completed easily and quickly (in about 14 minutes). The questions appeared to be relevant, reasonable, unambiguous, and clear. Therefore, face validity was considered to be very good. The content of the Greek version of EBPRS includes, in a balanced way, the full scope of the characteristics of nurses’ readiness towards EBP that it is intended to measure. These comprise identifying specifically attitudes towards EBP, perceived knowledge towards EBP, workplace EBP culture, informational needs, and EBP assessments of midwifery staff.

### Construct validity

Construct validity requires that there is no ‘cross loading’, i.e. it is not permitted in confirmatory factor analysis for one item to be loaded on the factor of a different subscale. Also, one factor loads on at least three variables.

Cronbach’s α was calculated for each of the following subscales of the Greek version of the EBPRS, with the questions, indicated in brackets, constituting the items for each subscale (Table 3):

1. EBP-attitude (MATES): (42, 44, 46, 49, 50, 53–58), α=0.561
2. Informational needs: (13, 18, 19, 29, 30), α=0.828
3. Workplace culture: (36–41), α=0.813
4. EBP-knowledge: (60, 61, 65, 66), α=0.493
5. EBP-assessments: (67–79), α=0.767

## DISCUSSION

The Greek version of EBPRS is a 39-item user-friendly and easy to complete self-report questionnaire, measuring nurses’ informational needs, workplace culture, EBP-attitude, perceived EBP-knowledge, and EBP assessments.

In this study, the concurrent, face, and content validity of the Greek EBPRS were grounded on quality assurance of the translation. Values of the standardized Cronbach’s alpha for the Greek EBPRS were found to be similar to those reported by Thiel and Ghosh^19^ in the first validation study. In general, in the present study, the majority of midwives were not familiar with EBP and indicated reading articles and books, attending congresses, seminars and educational programs, as well as asking questions to colleagues or fellow students, or having discussions with colleagues, as preferred methods of information seeking.

In the validation study of EBPRS in the US, the subscales MATES and EBP-knowledge had strong internal reliability with Cronbach’s alpha coefficient. In the present study, the Greek version of MATES is an 11-item tool with a Cronbach’s alpha coefficient; the English language version consists of 11 items. Thiel and Ghosh^19^ support the fact that EFA revealed eleven items load to one single factor (EBP-attitude). In the Greek version of MATES, EFA also reveals one single factor (EBP-attitude), with only 11-items loading on and one more single factor (EBP-assessments) with 13-items loading one. In the Greek version of EBP-knowledge, Cronbach’s alpha was calculated and had a value that was similar to the English-language tool and the relevant EBP-assessments value. In the present study, EFA revealed one single factor (EBP-knowledge) with four items loading on; in the English-language tool, one factor was identified with only three loading items. CFA showed that the five-factor model tested offered the desired fit to our data, as fit indices confirm.

Also, EBP-attitude was positively correlated both with ‘Informational needs’ and ‘Workplace culture’, suggesting that having adequate skills in information-seeking, and working in a setting with high EBP-culture, results in a positive attitude toward EBP. This also accords with our earlier findings, which showed that EBP-attitude is positively linked with a high educational level, individual skills, and positive organizational context^27-29^.

Furthermore, EBP-knowledge was positively correlated both with ‘Informational needs’ and ‘Workplace culture’, indicating that a midwife can adopt EBP-knowledge through developing adequate skills in a workplace that embraces EBP. This finding is supported by researchers indicating that EBP-knowledge is strongly correlated with level of skills and local organization’s culture^29^.

It is worth noting that Shaneyfelt et al.^30^ conducted a systematic review, presenting the EBP instruments used in education, revealing a large number of unique instruments (n=104) evaluating EBP. In particular, according to their findings, these tools explore EBP-knowledge (n=39), EBP-skills (n=59), EBP-attitudes (n=27), and EBP-behaviors (n=39). To our knowledge, the EBPRS is the only questionnaire that evaluates readiness towards EBP adoption by combining four different vital domains: EBP-attitude, Workplace culture, Informational needs, and EBP-knowledge.

Additionally, in international literature there is a small number of studies investigating the readiness of staff towards EBP using different research tools. Pravikoff et al.^31^ conducted a cross-sectional study in 1037 US nurses in order to study their readiness in EBP, focusing on their informational needs. They used ‘the information literacy for evidence-based nursing practice’ questionnaire, a small number of items of which have been included in EBPRS. The ‘EBP-preparedness of Australian nurses’ has been identified by Waters et al.^32^ using the ‘nurse's perceptions of EBP’ survey focusing on EBP attitudes and nurses’ EBP skills. Also, in the US, Gale and Schaffer^33^ studied the organizational readiness towards EBP using ‘EBP changes survey’ and highlighted the barriers and the reasons of preferring EBP integration.

So far, there is no evidence for midwifery personnel’s readiness towards EBP in Greek settings. The literature presents only two studies on EBP. The first study investigated the EBP attitudes among Greek health providers^34^. In this study, ‘EBP attitudes scale’ was completed by 604 physicians and only 70 nurses, which were finally excluded from the initial sample without providing any information about Greek nurses’ EBP attitudes. The second study assessed the readiness of nurses for the adoption of EBP^28^.

In this study, the sample consisted of 477 nurses working in five public hospitals and used the Greek version of the EBPRS adapted to nursing specialty in order to highlight the gap between practice and knowledge. Therefore, no study to date has focused on EBP midwives. Thus, this study, which validated the Greek version of EBPRS is a basis for the implementation of the EBP in the future regarding midwives. Similar to Patelarou et al.^28^, this study aims to identify and eliminate the gap between practice and knowledge, with a view to making best the quality of healthcare.

### Strengths and limitations

The EBPRS is the only questionnaire that encompasses five different vital domains (Informational needs, Workplace culture, EBP-knowledge, EBP-attitude, and EBP-assessments) to assess the readiness of midwives to EBP. Additionally, no previous studies were identified to translate and validate an EBP tool on a sample of Greek midwifery staff. On the other hand, one drawback of the methodology of this study is the self-reported information, which can raise problems of bias. However, both the sample size of the study and the method of randomization used for the data collection diminish the aforementioned limitations.

The main limitation of the research is that the sample consists of both female and male midwives as well as female students where some of the students have completed only the first year of study and therefore have very limited experience in the clinical setting of EBP. However, midwifery students learn from their first steps in obstetrics to rely on documented knowledge and are encouraged to search for information on relevant electronic databases, to visit the University Library frequently, and to attend conferences both as audience and speakers. Also, in all semesters, it is mandatory to write small papers that aim to familiarize students with finding substantiated knowledge, while in the last semester of studies (8th semester) they are encouraged to prepare a research dissertation. The Department of Midwifery of the University of Athens gives graduate midwives the opportunity to continue their studies with a postgraduate or doctoral program aimed at vocational training, research experience, specialization in the different fields, continuous information on new data, and lifelong education. Finally, the present survey included midwives, which implies that one should be very cautious when translating these results to other healthcare professions.

### Future research

Further research to cover diverse health professions and disciplines will emerge as future research priorities. In the Greek validation study for nursing, the original additional 66 items on the EBPRS did not demonstrate satisfactory levels of statistical validity and so were removed, which trimmed the questionnaire from a 66-item to a 23-item questionnaire. In this study was added one extra part which consists of 14 questions. On the other hand, according to validation Greek EBPRS for the midwives’ study, 39 of 80 items were excluded from the factor analysis, which were not important for Greek midwives. More specifically, exploratory factor analysis and confirmatory factor analysis showed that the 41 items as well as 14 extra part questions were useful for the Greek EBPRS. These questions, which were removed, seem to relate to the way of seeking information provided at the workplace. It is worth mentioning that many of the questions removed were common in the two studies.

## CONCLUSIONS

The Greek version of the EBPRS appears to be a reliable and valid tool, being quick and easy to complete. This instrument may become a useful tool in future research assessing midwifery readiness towards EBP implementation and focusing on the nursing staff vital domains of: EBP-attitude, Informational needs, Workplace culture, EBP-knowledge, and EBP-assessments. It is evident that this study provides information that addresses the lacuna in the literature referring to the readiness of the Greek midwives for the adoption of EBP. For a successful implementation of EBP in everyday midwifery practice, it is a requirement for the personnel to be trained in order to develop the needed skills and abilities, so that they can be efficient at searching in the respective data sources. In addition, the midwifery personnel must be encouraged to take part in the decision-making process as well as in other relevant initiatives that would ultimately promote the use of EBP. Another factor that would increase awareness would be for EBP-related topics to be more actively included in the basic midwifery curricula.

## References

[1] Meah S, Luker KA, Cullum NA (1996). An exploration of midwives' attitudes to research and perceived barriers to research utilisation. Midwifery.

[2] Burgum M (1997). Promoting EBP in midwifery (evidencebased practice). Aust Nurs J.

[3] Albers LL (2001). Evidence and midwifery practice. J Midwifery Womens Health.

[4] Veeramah V (2004). Utilization of research findings by graduate nurses and midwives. J Adv Nurs.

[5] Sackett DL, Rosenberg WMC, Gray JAM, Haynes RB, Richardson WS (1996). Evidence based medicine: What it is and what it isn’t. BMJ.

[6] Elldrodt G, Cook DJ, Lee J, Cho M, Hunt D, Weingarten S (1997). Evidence-based disease management. JAMA.

[7] Bick D (2011). Evidence based midwifery practice: Take care to 'mind the gap'. Midwifery.

[8] Ericson-Owens DA, Kennedy HP (2001). Fostering evidence based care in clinical teaching. J Midwifery Womens Health.

[9] Lange G, Kennedy HP (2006). Student Perceptions of Ideal and Actual Midwifery Practice. J Midwifery Womens Health.

[10] Thorsteinsson HS (2013). Icelandic Nurses’ Beliefs, Skills, and Resources Associated with Evidence-Based Practice and Related Factors: A National Survey. Worldviews Evid Based Nurs.

[11] Brownson RC, Chriqui JF, Stamatakis KA (2009). Understanding evidence-based public health policy. Am J Public Health.

[12] King JF (2005). A short history of evidence-based obstetric care. Best Pract Res Clin Obstet Gynaecol.

[13] Hunter LP, Lops VR (1994). Critical thinking and the nurse-midwifery management process. J Nurse Midwifery.

[14] Pravikoff DS, Tanner AB, Pierce ST (2005). Readiness of U.S. nurses for evidence-based practice. Am J Nurs.

[15] Bogdan-Lovis EA, Sousa A (2006). The Contextual Influence of Professional Culture: Certified Nurse-Midwives’ Knowledge of and Reliance on Evidence-Based Practice. Soc Sci Med.

[16] Hayes E (1994). Helping preceptors mentor the next generation of nurse practitioners. Nurse Pact.

[17] Kyriakoulis K, Patelarou A, Laliotis A (2016). Educational strategies for teaching evidence-based practice to undergraduate health students systematic review. J Educ Eval Health Prof.

[18] Titler MG, Hill J, Matthew G, Reed D Development and validation of an instrument to measure barriers to research utilization.

[19] Thiel L, Ghosh Y (2008). Determining registered nurses' readiness for evidence based practice. Worldviews Evid Based Nurs.

[20] Kline RB (2005). Principles and practice of structural equation modeling.

[21] De Vellis RF (1991). Scale Development: Theory and applications.

[22] de Vet HC, Bouter LM, Bezemer PD, Beurskens AJ (2001). Reproducibility and responsiveness of evaluative outcome measures. Theoretical considerations illustrated by an empirical example. Int J Technol Assess Health Care.

[23] Tabachnick BG, Fidell LS (2007). Using Multivariate Statistics.

[24] Hakstian AR, Rogers WΤ, Cattell RB (1982). The Behavior Of Number-Of-Factors Rules With Simulated Data. Multivariate Behav Res.

[25] Morrison DF (1976). Multivariate Statistical Methods.

[26] Kaiser HF (1960). The application of electronic computers to factor analysis. Educ Psychol Meas.

[27] Stahmer AC, Aarons G (2009). Attitudes Toward Adoption of Evidence-Based Practices: A comparison of Autism Early Intervention Providers and Children's Mental Health Providers. Psychol Serv.

[28] Patelarou AE, Dafermos V, Brokalaki H, Melas C, Koukia E (2014). Readiness toward evidence-based practice implementation; Can it be measured?. Perioriative Nursing.

[29] Melnyk BM, Fineout-Overholt E, Stillwell SB, Williamson KM (2009). Evidence-based practice: step by step: igniting a spirit of inquiry: an essential foundation for evidence-based practice. American J Nurs.

[30] Shaneyfelt T, Baum KD, Bell D (2006). Instruments for evaluating education in evidence-based practice: A systematic review. JAMA.

[31] Pravikoff DS, Tanner AB, Pierce ST (2005). Readiness of U.S. nurses for evidence-based practice. Am J Nurs.

[32] Waters D, Crisp J, Rychetnik L, Barratt A (2009). The Australian experience of nurses' preparedness for evidence-based practice. J Nurs Manag.

[33] Gale BVP, Schaffer MA (2009). Organizational readiness for evidence-based practice. J Nurs Adm.

[34] Melas CD, Zampetakis LA, Dimopoulou A, Moustakis V (2012). Evaluating the Properties of the Evidence-Based Practice Attitude Scale (EBPAS) in Health Care. Psychol Assess.

