# Supplementary material for: A survey for the readiness of Greek midwives for the adoption of evidence-based practice (EBP)
Source: Eur J Midwifery. 2020 Nov 20;4:43. doi: 10.18332/ejm/128270 (PMC7839138; doi:10.18332/ejm/128270)
Supplement: Supplementary file 1 [file EJM-4-43-s1.pdf]

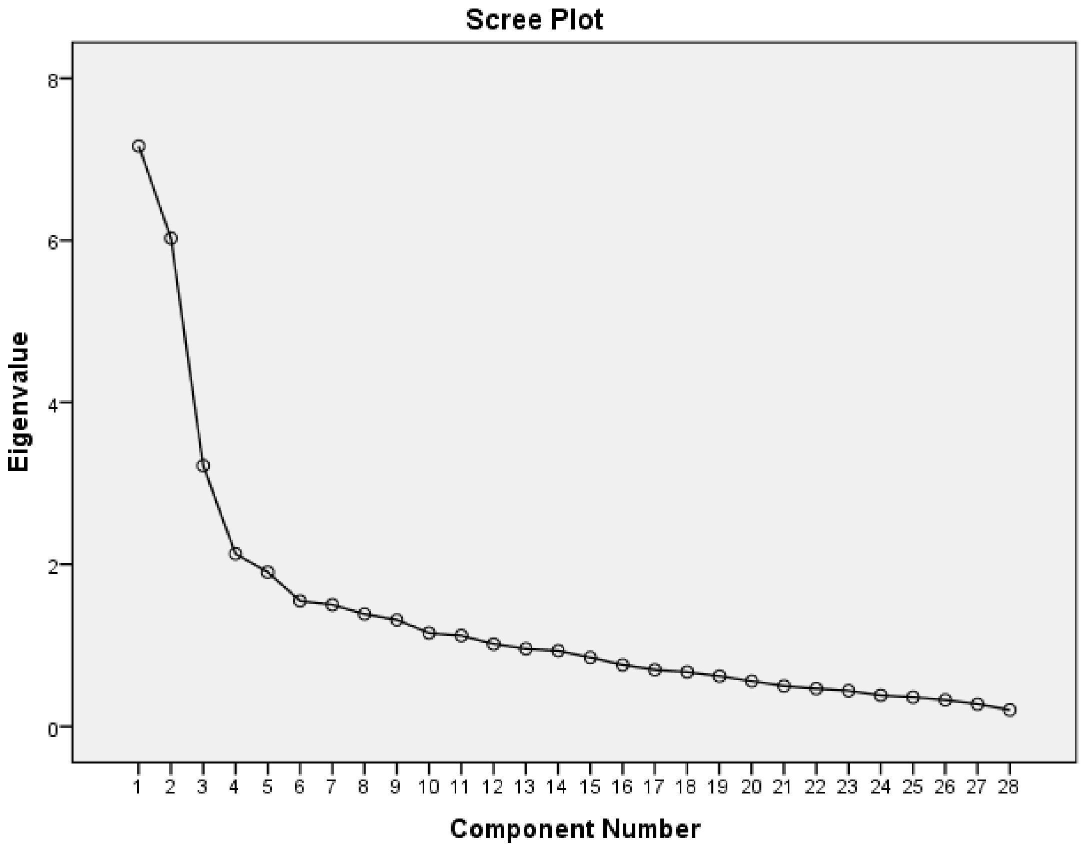

Figure 1. Scree plot

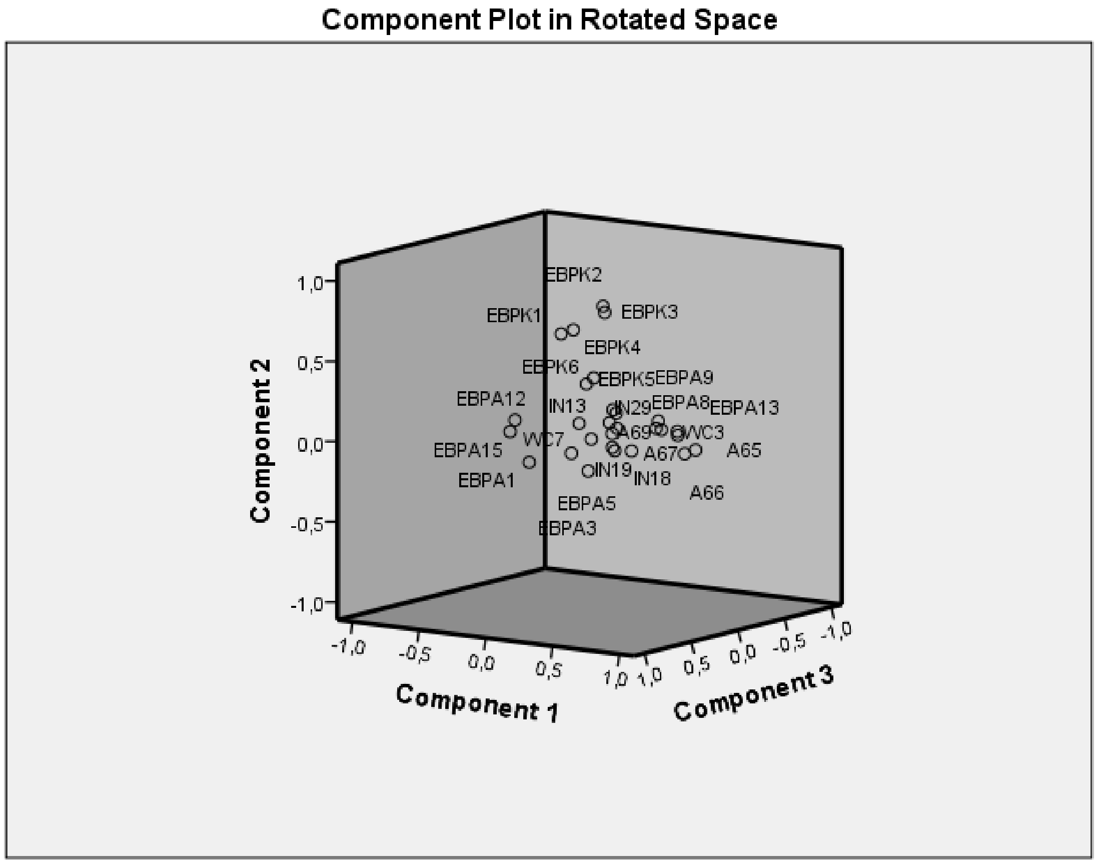

Figure 2. Component plot in rotated space

TABLE S1. Descriptive Statistics of Items of the Greek EBPRS

**Items***		**Mean**	**SD**	**Skewness**	**Kurtosis**
**13**	Κάνω αναζήτηση σε βιβλιογραφικές βάσεις δεδομένων.	2.47	1.221	0.502	-0.576
**18**	Βαθμός ικανότητας για αναζήτηση πληροφορίας στην βιβλιογραφική βάση δεδομένων CINAHL.	3.84	1.302	-0.883	-0.202
**19**	Βαθμός ικανότητας για αναζήτηση πληροφορίας στην βιβλιογραφική βάση δεδομένων MEDLINE/PUBMED.	3.39	1.434	-0.389	-1.094
**29**	Συχνότητα αναζήτησης πληροφορίας στη CINAHL.	3.96	1.526	-1.123	-0.351
**30**	Συχνότητα αναζήτησης πληροφορίας στη MEDLINE/PUBMED.	3.53	1.641	-0.594	-1.328
**36**	Οι μαίες/μαιευτές με τους οποίους συνεργάζομαι θα δήλωναν ότι: Υπάρχει καλό επίπεδο συνεργασίας μεταξύ των μαιών/μαιευτών.	2.89	1.203	0.357	-0.319
**37**	Οι μαίες/μαιευτές με τους οποίους συνεργάζομαι θα δήλωναν ότι: Είναι ικανοποιημένοι με την αλληλεπίδραση μεταξύ των μαιών/μαιευτών.	3.16	1.178	0.001	-0.122
**38**	Οι μαίες/μαιευτές με τους οποίους συνεργάζομαι θα δήλωναν ότι: Οι γιατροί γενικά συνεργάζονται με τις μαίες/τους μαιευτές.	3.30	1.373	-0.096	-0.295
**39**	Οι μαίες/μαιευτές με τους οποίους συνεργάζομαι θα δήλωναν ότι: Υπάρχει έντονη ομαδικότητα μεταξύ μαιών/μαιευτών και ιατρών στο τμήμα αυτό..	3.22	1.420	-0.142	-0.589
**40**	Οι μαίες/μαιευτές με τους οποίους συνεργάζομαι θα δήλωναν ότι: Δίνονται συχνά ευκαιρίες στις μαίες/ στους μαιευτές να συμμετέχουν στη λήψη αποφάσεων όσον αφορά την κλινική πράξη.	3.59	1.429	-0.065	-0.785
**41**	Οι μαίες/μαιευτές με τους οποίους συνεργάζομαι θα δήλωναν ότι: Μπορούν να εκφέρουν την άποψή τους στο βαθμό που θα ήθελαν όσον αφορά στο σχεδιασμό πολιτικών και διαδικασιών στο τμήμα αυτό.	3.67	1.477	-0.093	-0.889
**42**	Προσωπικά δεν έχω κανένα λόγο να υιοθετήσω την τεκμηριωμένη λήψη κλινικής απόφασης (ΤΛΚΑ), επειδή είναι μια μόδα που θα περάσει με το χρόνο.	3.61	1.135	-0.718	0.277
**44**	Η ΤΛΚΑ αγνοεί την κλινική εμπειρία.	3.38	0.984	-0.433	0.254
**46**	Η ΤΛΚΑ αγνοεί την «τέχνη» της μαιευτικής.	3.46	1.096	-0.648	0.659
**49**	Έχω αρκετές γνώσεις προκειμένου να ασχοληθώ με την ΤΛΚΑ.	3.31	1.136	-1.280	2.082
**50**	Έχω αρκετή πρόσβαση σε πηγές προκειμένου να ασχοληθώ με την ΤΛΚΑ.	2.18	0.884	0.658	0.461
**53**	Οι μαίες/τες γενικά δεν θα έπρεπε να εφαρμόζουν την ΤΛΚΑ δεδομένου ότι η μαιευτική έχει να κάνει με την γυναίκα, όχι με τη στατιστική.	3.45	1.130	-0.682	0.240
**54**	Είμαι βέβαιος/-η ότι μπορώ να ασχοληθώ με την ΤΛΚΑ .	2.74	0.862	-0,418	1.131
**55**	Έχω αρκετές ικανότητες προκειμένου να ασχοληθώ με την ΤΛΚΑ.	2.87	0.974	-0.745	1.631
**56**	Έχω αρκετές ικανότητες προκειμένου να ασχοληθώ με την ΤΛΚΑ.	3.52	1.043	-0.790	1.106
**57**	Η εφαρμογή της ΤΛΚΑ αυξάνει την πιθανότητα να επιτευχθούν τα	2.33	0.936	0.326	0.352

	επιθυμητά για τον ασθενή αποτελέσματα..				
**58**	Είναι σημαντικό ότι στο νοσοκομείο μας υιοθετείται η πρακτική βασισμένη σε ενδείξεις στη μαιευτική πρακτική.	2.62	1.112	-0.099	-0.260
**60**	Γνωρίζω για τη μαιευτική έρευνα από συζητήσεις με συναδέλφους μου.	2.79	1.080	0.174	-0.351
**61**	Έχω εύκολη πρόσβαση σε μαιευτικά ερευνητικά άρθρα.	2.76	1.079	-0.114	-0.683
**65**	Μαίες/τες, όπως είναι π.χ. οι κλινικοί εκπαιδευτές, δρουν ως μέντορες στο τμήμα μου.	2.74	1.039	0.156	-0.179
**66**	Μπορώ να διαβάσω μια μαιευτική έρευνα και να ασκήσω μια εν τω βάθει κριτική για την επιστημονική του αξία.	2.80	0.874	0.045	0.335
**67**	Η ΤΛΚΑ συμβάλλει στην οικονομικότερη μαιευτική φροντίδα.	2.48	0.779	-0.344	-0.414
**68**	Η ΤΛΚΑ βοηθάει στην διάθεση των οικονομικών πόρων με αποτελεσματικότερο τρόπο.	2.55	0.713	-0.282	0.294
**69**	Η ΤΛΚΑ αυξάνει την αποδοτικότητα των μαιών/μαιευτών.	2.34	0.812	-0.112	-0.105
**70**	Η ΤΛΚΑ προσφέρει στην μονάδα υγείας επιστημονική κατοχύρωση.	2.16	0.887	-0.232	0.192
**71**	Η δύναμη της συνήθειας και της ρουτίνας στην κλινική πράξη παρεμποδίζει την εφαρμογή της ΤΛΚΑ.	2.37	0.958	0.052	-0.398
**72**	Η έλλειψη αυτοπεποίθησης για την ικανότητα εφαρμογής νέων κλινικών μεθόδων δυσκολεύει την εφαρμογή της ΤΛΚΑ.	2.38	0.904	0.404	-0.062
**73**	Νιώθω ικανή να εφαρμόσω στην μαιευτική φροντίδα την ΤΛΚΑ.	2.70	0.876	-0.206	0.572
**74**	Εχω τις γνώσεις να εφαρμόσω την ΤΛΚΑ στην κλινική πρακτική.	2.83	0.985	-0.806	1.323
**75**	Έχω εμπιστοσύνη στον εαυτό μου για την αξιολόγηση της κατάστασης των γυναικών που φροντίζω.	2.15	0.921	0.596	0.787
**76**	Έχω εμπιστοσύνη στον εαυτό μου για τις μεθόδους που εφαρμόζω στην μαιευτική φροντίδα.	2.03	0.828	0.245	0.425
**77**	Μπορώ να αξιολογήσω την εγκυρότητα των στοιχείων μιας έρευνας.	2.51	0.899	-0.302	0.710
**78**	Μπορώ να εφαρμόσω τα νέα ερευνητικά δεδομένα στην κλινική πράξη.	2.63	0.822	-0.376	0.713
**79**	Η αξιολόγηση της κλινικής εργασίας μου σχετίζεται άμεσα με την ΤΛΚΑ.	2.75	0.965	-0.478	0.418

Table S2. Inter-item Correlation Matrix for the Greek EBPRS

ITEM	13	18	19	29	30	36	37	38	39	40	41	42	44	46	49	50	53	54	55	56	57	58	60	61	65	66	67	68	69	70	71	72	73	74	75	76	77	78	79
**13**	1,00	0,29	0,42	0,28	0,36	0,06	-0,03	-0,06	-0,11	0,03	0,00	-0,16	-0,02	0,01	0,07	0,13	-0,05	0,17	0,16	-0,01	0,19	0,12	0,03	0,19	0,06	0,19	-0,02	0,06	0,25	0,11	0,23	0,15	0,02	0,10	-0,02	-0,05	0,14	0,16	0,07
**18**	0,29	1,00	0,74	0,58	0,45	0,12	-0,04	-0,02	-0,08	0,07	0,07	0,02	-0,03	0,07	0,08	0,06	0,01	0,24	0,15	0,03	0,03	0,17	0,03	0,30	0,06	0,21	0,20	0,18	0,20	0,14	0,12	0,08	0,21	0,19	0,08	0,15	0,26	0,19	0,22
**19**	0,42	0,74	1,00	0,45	0,64	0,04	-0,08	-0,08	-0,18	0,04	0,02	-0,11	-0,13	0,03	0,06	0,13	-0,05	0,31	0,27	-0,02	0,15	0,22	-0,06	0,28	0,08	0,23	0,17	0,16	0,26	0,25	0,21	0,22	0,20	0,13	0,03	0,07	0,17	0,17	0,19
**29**	0,28	0,58	0,45	1,00	0,67	0,11	0,03	0,01	0,01	0,10	0,14	-0,02	-0,07	0,07	0,10	0,10	0,00	0,29	0,22	0,05	0,07	0,20	0,02	0,24	0,12	0,26	0,05	0,12	0,20	0,16	0,20	0,01	0,15	0,25	0,09	0,10	0,19	0,13	0,28
**30**	0,36	0,45	0,64	0,67	1,00	0,04	0,02	-0,04	-0,07	0,00	0,03	-0,17	-0,18	-0,02	0,08	0,25	-0,04	0,27	0,32	-0,03	0,13	0,18	-0,04	0,18	0,15	0,24	0,08	0,16	0,27	0,26	0,18	0,16	0,17	0,22	0,04	0,10	0,14	0,16	0,27
**36**	0,06	0,12	0,04	0,11	0,04	1,00	0,60	0,31	0,29	0,31	0,32	0,07	0,02	-0,05	0,02	-0,10	0,16	0,11	-0,09	0,14	-0,02	0,08	-0,02	-0,01	0,05	0,08	0,01	-0,01	-0,06	-0,14	-0,04	-0,14	-0,08	-0,02	0,02	0,01	0,11	-0,05	-0,04
**37**	-0,03	-0,04	-0,08	0,03	0,02	0,60	1,00	0,46	0,47	0,41	0,42	-0,08	-0,05	-0,06	-0,02	0,00	0,04	0,13	0,04	0,03	0,05	0,04	0,13	0,14	0,08	0,11	-0,08	0,02	-0,05	-0,11	-0,16	-0,10	0,04	0,03	0,01	-0,02	0,13	-0,11	-0,05
**38**	-0,06	-0,02	-0,08	0,01	-0,04	0,31	0,46	1,00	0,43	0,46	0,41	0,06	0,02	-0,01	0,11	0,02	0,03	-0,03	-0,09	-0,02	0,04	0,06	0,10	0,01	-0,08	-0,04	-0,12	-0,04	-0,05	-0,10	-0,16	-0,04	-0,05	-0,06	0,06	-0,02	0,02	-0,01	0,08
**39**	-0,11	-0,08	-0,18	0,01	-0,07	0,29	0,47	0,43	1,00	0,39	0,32	0,00	0,00	-0,03	-0,02	-0,10	0,09	0,08	-0,13	0,06	-0,05	0,16	0,17	0,00	0,13	0,01	-0,02	0,01	-0,05	-0,09	-0,13	-0,10	-0,07	-0,05	-0,08	0,03	0,03	0,07	0,05
**40**	0,03	0,07	0,04	0,10	0,00	0,31	0,41	0,46	0,39	1,00	0,73	0,23	0,02	0,15	0,11	-0,13	-0,01	0,13	-0,01	0,10	-0,04	0,14	0,05	0,11	0,09	0,08	0,10	0,04	-0,06	-0,06	-0,02	-0,12	0,00	0,02	0,09	0,10	0,15	0,05	-0,02
**41**	0,00	0,07	0,02	0,14	0,03	0,32	0,42	0,41	0,32	0,73	1,00	0,15	0,06	0,18	0,08	-0,10	0,05	0,09	-0,03	0,13	-0,07	0,12	0,01	0,04	-0,01	0,12	0,04	-0,07	-0,11	-0,08	-0,07	-0,17	-0,02	0,06	0,09	0,08	0,05	-0,02	0,02
**42**	-0,16	0,02	-0,11	-0,02	-0,17	0,07	-0,08	0,06	0,00	0,23	0,15	1,00	0,44	0,32	0,31	-0,27	0,33	-0,07	0,02	0,26	-0,30	-0,02	0,01	-0,06	-0,02	-0,08	0,08	-0,08	-0,14	-0,18	-0,09	-0,16	-0,19	-0,01	-0,10	0,04	0,03	0,04	0,02
**44**	-0,02	-0,03	-0,13	-0,07	-0,18	0,02	-0,05	0,02	0,00	0,02	0,06	0,44	1,00	0,41	0,32	-0,23	0,24	-0,14	-0,06	0,20	-0,24	-0,06	0,04	0,05	0,00	-0,03	-0,04	-0,07	-0,22	-0,10	-0,05	-0,09	0,00	0,12	0,02	-0,03	0,10	-0,04	0,10
**46**	0,01	0,07	0,03	0,07	-0,02	-0,05	-0,06	-0,01	-0,03	0,15	0,18	0,32	0,41	1,00	0,33	-0,27	0,10	0,07	0,01	0,17	-0,21	0,06	0,01	0,14	-0,05	0,06	0,01	-0,13	-0,16	-0,01	0,08	-0,16	-0,01	0,11	-0,01	0,02	0,16	0,01	-0,02
**49**	0,07	0,08	0,06	0,10	0,08	0,02	-0,02	0,11	-0,02	0,11	0,08	0,31	0,32	0,33	1,00	-0,13	0,14	0,01	-0,02	0,27	-0,08	0,10	0,02	0,13	-0,04	-0,09	-0,11	-0,09	-0,02	-0,08	0,07	-0,01	-0,05	0,03	0,04	-0,04	0,09	-0,02	0,05
**50**	0,13	0,06	0,13	0,10	0,25	-0,10	0,00	0,02	-0,10	-0,13	-0,10	-0,27	-0,23	-0,27	-0,13	1,00	-0,26	0,32	0,26	-0,29	0,35	0,08	-0,06	0,06	0,09	0,01	0,10	0,17	0,36	0,33	0,10	0,31	0,11	0,14	0,10	-0,03	0,06	-0,03	0,12
**53**	-0,05	0,01	-0,05	0,00	-0,04	0,16	0,04	0,03	0,09	-0,01	0,05	0,33	0,24	0,10	0,14	-0,26	1,00	-0,16	-0,10	0,37	-0,22	0,14	0,14	-0,04	-0,05	0,02	-0,12	-0,14	-0,15	-0,25	-0,02	-0,09	-0,11	-0,01	-0,08	-0,06	-0,02	-0,07	-0,03
**54**	0,17	0,24	0,31	0,29	0,27	0,11	0,13	-0,03	0,08	0,13	0,09	-0,07	-0,14	0,07	0,01	0,32	-0,16	1,00	0,39	-0,14	0,20	0,16	0,02	0,25	0,15	0,15	0,23	0,17	0,29	0,19	0,25	0,12	0,25	0,21	0,14	0,07	0,22	0,05	0,05
**55**	0,16	0,15	0,27	0,22	0,32	-0,09	0,04	-0,09	-0,13	-0,01	-0,03	0,02	-0,06	0,01	-0,02	0,26	-0,10	0,39	1,00	-0,04	0,15	0,11	0,10	0,14	0,03	0,27	0,17	0,19	0,11	0,17	0,13	0,25	0,20	0,10	0,00	-0,04	0,06	0,04	0,14
**56**	-0,01	0,03	-0,02	0,05	-0,03	0,14	0,03	-0,02	0,06	0,10	0,13	0,26	0,20	0,17	0,27	-0,29	0,37	-0,14	-0,04	1,00	-0,10	0,13	0,02	-0,01	-0,04	0,07	-0,16	-0,08	-0,27	-0,21	-0,11	-0,23	-0,16	-0,14	-0,09	0,02	-0,02	-0,06	-0,06
**57**	0,19	0,03	0,15	0,07	0,13	-0,02	0,05	0,04	-0,05	-0,04	-0,07	-0,30	-0,24	-0,21	-0,08	0,35	-0,22	0,20	0,15	-0,10	1,00	0,24	0,04	-0,04	0,23	-0,05	0,10	0,26	0,32	0,21	0,04	0,17	0,00	-0,10	-0,09	-0,17	0,04	0,05	0,02
**58**	0,12	0,17	0,22	0,20	0,18	0,08	0,04	0,06	0,16	0,14	0,12	-0,02	-0,06	0,06	0,10	0,08	0,14	0,16	0,11	0,13	0,24	1,00	0,10	0,02	0,25	0,07	-0,02	-0,13	0,13	0,08	0,22	0,14	0,04	0,06	-0,06	0,03	0,12	0,13	0,04
**60**	0,03	0,03	-0,06	0,02	-0,04	-0,02	0,13	0,10	0,17	0,05	0,01	0,01	0,04	0,01	0,02	-0,06	0,14	0,02	0,10	0,02	0,04	0,10	1,00	0,19	0,18	0,10	0,09	0,09	0,12	0,00	-0,11	-0,07	0,12	0,11	-0,04	-0,01	0,16	0,14	0,12

**61**	0,19	0,30	0,28	0,24	0,18	-0,01	0,14	0,01	0,00	0,11	0,04	-0,06	0,05	0,14	0,13	0,06	-0,04	0,25	0,14	-0,01	-0,04	0,02	0,19	1,00	0,04	0,27	0,02	0,09	0,09	0,15	0,17	0,13	0,29	0,34	0,18	0,16	0,32	0,20	0,05
**65**	0,06	0,06	0,08	0,12	0,15	0,05	0,08	-0,08	0,13	0,09	-0,01	-0,02	0,00	-0,05	-0,04	0,09	-0,05	0,15	0,03	-0,04	0,23	0,25	0,18	0,04	1,00	0,07	0,13	0,07	0,15	0,20	0,07	0,03	0,06	0,07	-0,12	-0,11	0,09	0,09	0,12
**66**	0,19	0,21	0,23	0,26	0,24	0,08	0,11	-0,04	0,01	0,08	0,12	-0,08	-0,03	0,06	-0,09	0,01	0,02	0,15	0,27	0,07	-0,05	0,07	0,10	0,27	0,07	1,00	0,23	0,18	0,14	0,28	0,27	0,03	0,26	0,21	0,10	0,24	0,34	0,28	0,14
**67**	-0,02	0,20	0,17	0,05	0,08	0,01	-0,08	-0,12	-0,02	0,10	0,04	0,08	-0,04	0,01	-0,11	0,10	-0,12	0,23	0,17	-0,16	0,10	-0,02	0,09	0,02	0,13	0,23	1,00	0,66	0,42	0,42	0,16	0,21	0,21	0,07	0,08	0,26	0,30	0,20	0,21
**68**	0,06	0,18	0,16	0,12	0,16	-0,01	0,02	-0,04	0,01	0,04	-0,07	-0,08	-0,07	-0,13	-0,09	0,17	-0,14	0,17	0,19	-0,08	0,26	-0,13	0,09	0,09	0,07	0,18	0,66	1,00	0,43	0,32	0,08	0,27	0,16	0,02	0,04	0,17	0,26	0,15	0,15
**69**	0,25	0,20	0,26	0,20	0,27	-0,06	-0,05	-0,05	-0,05	-0,06	-0,11	-0,14	-0,22	-0,16	-0,02	0,36	-0,15	0,29	0,11	-0,27	0,32	0,13	0,12	0,09	0,15	0,14	0,42	0,43	1,00	0,51	0,24	0,26	0,22	0,12	0,12	0,08	0,26	0,12	0,23
**70**	0,11	0,14	0,25	0,16	0,26	-0,14	-0,11	-0,10	-0,09	-0,06	-0,08	-0,18	-0,10	-0,01	-0,08	0,33	-0,25	0,19	0,17	-0,21	0,21	0,08	0,00	0,15	0,20	0,28	0,42	0,32	0,51	1,00	0,27	0,29	0,18	0,16	0,02	0,06	0,20	0,16	0,27
**71**	0,23	0,12	0,21	0,20	0,18	-0,04	-0,16	-0,16	-0,13	-0,02	-0,07	-0,09	-0,05	0,08	0,07	0,10	-0,02	0,25	0,13	-0,11	0,04	0,22	-0,11	0,17	0,07	0,27	0,16	0,08	0,24	0,27	1,00	0,25	0,03	0,14	0,22	0,07	0,12	0,04	0,03
**72**	0,15	0,08	0,22	0,01	0,16	-0,14	-0,10	-0,04	-0,10	-0,12	-0,17	-0,16	-0,09	-0,16	-0,01	0,31	-0,09	0,12	0,25	-0,23	0,17	0,14	-0,07	0,13	0,03	0,03	0,21	0,27	0,26	0,29	0,25	1,00	0,11	0,05	0,00	-0,10	-0,03	0,06	0,06
**73**	0,02	0,21	0,20	0,15	0,17	-0,08	0,04	-0,05	-0,07	0,00	-0,02	-0,19	0,00	-0,01	-0,05	0,11	-0,11	0,25	0,20	-0,16	0,00	0,04	0,12	0,29	0,06	0,26	0,21	0,16	0,22	0,18	0,03	0,11	1,00	0,44	0,37	0,28	0,41	0,26	0,31
**74**	0,10	0,19	0,13	0,25	0,22	-0,02	0,03	-0,06	-0,05	0,02	0,06	-0,01	0,12	0,11	0,03	0,14	-0,01	0,21	0,10	-0,14	-0,10	0,06	0,11	0,34	0,07	0,21	0,07	0,02	0,12	0,16	0,14	0,05	0,44	1,00	0,38	0,22	0,23	0,26	0,13
**75**	-0,02	0,08	0,03	0,09	0,04	0,02	0,01	0,06	-0,08	0,09	0,09	-0,10	0,02	-0,01	0,04	0,10	-0,08	0,14	0,00	-0,09	-0,09	-0,06	-0,04	0,18	-0,12	0,10	0,08	0,04	0,12	0,02	0,22	0,00	0,37	0,38	1,00	0,53	0,24	0,15	0,14
**76**	-0,05	0,15	0,07	0,10	0,10	0,01	-0,02	-0,02	0,03	0,10	0,08	0,04	-0,03	0,02	-0,04	-0,03	-0,06	0,07	-0,04	0,02	-0,17	0,03	-0,01	0,16	-0,11	0,24	0,26	0,17	0,08	0,06	0,07	-0,10	0,28	0,22	0,53	1,00	0,36	0,27	0,18
**77**	0,14	0,26	0,17	0,19	0,14	0,11	0,13	0,02	0,03	0,15	0,05	0,03	0,10	0,16	0,09	0,06	-0,02	0,22	0,06	-0,02	0,04	0,12	0,16	0,32	0,09	0,34	0,30	0,26	0,26	0,20	0,12	-0,03	0,41	0,23	0,24	0,36	1,00	0,35	0,29
**78**	0,16	0,19	0,17	0,13	0,16	-0,05	-0,11	-0,01	0,07	0,05	-0,02	0,04	-0,04	0,01	-0,02	-0,03	-0,07	0,05	0,04	-0,06	0,05	0,13	0,14	0,20	0,09	0,28	0,20	0,15	0,12	0,16	0,04	0,06	0,26	0,26	0,15	0,27	0,35	1,00	0,33
**79**	0,07	0,22	0,19	0,28	0,27	-0,04	-0,05	0,08	0,05	-0,02	0,02	0,02	0,10	-0,02	0,05	0,12	-0,03	0,05	0,14	-0,06	0,02	0,04	0,12	0,05	0,12	0,14	0,21	0,15	0,23	0,27	0,03	0,06	0,31	0,13	0,14	0,18	0,29	0,33	1,00

© 2020 Ladopoulou A et al.
